# The centromeric gene *OsDCL* plays essential roles in plant development and yield production in rice

**DOI:** 10.1093/plphys/kiaf500

**Published:** 2025-10-29

**Authors:** Xiangbo Li, Chao Xue, Rui Guo, Zhiying Wang, Kai Liu, Mingchen Shen, Lu Ye, Jijin Chen, Hanli You, Jun Miao, Liang Chen, Wenhui Bian, Yong Zhou, Zhiyun Gong

**Affiliations:** Jiangsu Key Laboratory of Crop Genomics and Molecular Breeding/Zhongshan Biological Breeding Laboratory/Key Laboratory of Plant Functional Genomics of the Ministry of Education, Agricultural College of Yangzhou University, Yangzhou 225009, China; Jiangsu Co-Innovation Center for Modern Production Technology of Grain Crops/Jiangsu Key Laboratory of Crop Genetics and Physiology, Yangzhou 225009, China; Yangzhou Modern Seed Innovation Institute, Gaoyou 225600, China; Jiangsu Key Laboratory of Crop Genomics and Molecular Breeding/Zhongshan Biological Breeding Laboratory/Key Laboratory of Plant Functional Genomics of the Ministry of Education, Agricultural College of Yangzhou University, Yangzhou 225009, China; School of Agronomy and Horticulture, Jiangsu Vocational College of Agriculture and Forestry, Jurong 212400, China; Jiangsu Key Laboratory of Crop Genomics and Molecular Breeding/Zhongshan Biological Breeding Laboratory/Key Laboratory of Plant Functional Genomics of the Ministry of Education, Agricultural College of Yangzhou University, Yangzhou 225009, China; Jiangsu Co-Innovation Center for Modern Production Technology of Grain Crops/Jiangsu Key Laboratory of Crop Genetics and Physiology, Yangzhou 225009, China; Jiangsu Key Laboratory of Crop Genomics and Molecular Breeding/Zhongshan Biological Breeding Laboratory/Key Laboratory of Plant Functional Genomics of the Ministry of Education, Agricultural College of Yangzhou University, Yangzhou 225009, China; Jiangsu Co-Innovation Center for Modern Production Technology of Grain Crops/Jiangsu Key Laboratory of Crop Genetics and Physiology, Yangzhou 225009, China; Jiangsu Key Laboratory of Crop Genomics and Molecular Breeding/Zhongshan Biological Breeding Laboratory/Key Laboratory of Plant Functional Genomics of the Ministry of Education, Agricultural College of Yangzhou University, Yangzhou 225009, China; Jiangsu Key Laboratory of Crop Genomics and Molecular Breeding/Zhongshan Biological Breeding Laboratory/Key Laboratory of Plant Functional Genomics of the Ministry of Education, Agricultural College of Yangzhou University, Yangzhou 225009, China; Jiangsu Key Laboratory of Crop Genomics and Molecular Breeding/Zhongshan Biological Breeding Laboratory/Key Laboratory of Plant Functional Genomics of the Ministry of Education, Agricultural College of Yangzhou University, Yangzhou 225009, China; Jiangsu Co-Innovation Center for Modern Production Technology of Grain Crops/Jiangsu Key Laboratory of Crop Genetics and Physiology, Yangzhou 225009, China; Jiangsu Key Laboratory of Crop Genomics and Molecular Breeding/Zhongshan Biological Breeding Laboratory/Key Laboratory of Plant Functional Genomics of the Ministry of Education, Agricultural College of Yangzhou University, Yangzhou 225009, China; Jiangsu Co-Innovation Center for Modern Production Technology of Grain Crops/Jiangsu Key Laboratory of Crop Genetics and Physiology, Yangzhou 225009, China; Jiangsu Key Laboratory of Crop Genomics and Molecular Breeding/Zhongshan Biological Breeding Laboratory/Key Laboratory of Plant Functional Genomics of the Ministry of Education, Agricultural College of Yangzhou University, Yangzhou 225009, China; Jiangsu Key Laboratory of Crop Genomics and Molecular Breeding/Zhongshan Biological Breeding Laboratory/Key Laboratory of Plant Functional Genomics of the Ministry of Education, Agricultural College of Yangzhou University, Yangzhou 225009, China; Jiangsu Key Laboratory of Crop Genomics and Molecular Breeding/Zhongshan Biological Breeding Laboratory/Key Laboratory of Plant Functional Genomics of the Ministry of Education, Agricultural College of Yangzhou University, Yangzhou 225009, China; Jiangsu Co-Innovation Center for Modern Production Technology of Grain Crops/Jiangsu Key Laboratory of Crop Genetics and Physiology, Yangzhou 225009, China; Yangzhou Modern Seed Innovation Institute, Gaoyou 225600, China; Jiangsu Key Laboratory of Crop Genomics and Molecular Breeding/Zhongshan Biological Breeding Laboratory/Key Laboratory of Plant Functional Genomics of the Ministry of Education, Agricultural College of Yangzhou University, Yangzhou 225009, China; Jiangsu Co-Innovation Center for Modern Production Technology of Grain Crops/Jiangsu Key Laboratory of Crop Genetics and Physiology, Yangzhou 225009, China; Yangzhou Modern Seed Innovation Institute, Gaoyou 225600, China

## Abstract

The rice centromeric gene *OsDCL* boosts yield by regulating chlorophyll synthesis and photosynthesis, making it a key genetic resource for breeding high-yield rice with superior photosynthetic efficiency.

Dear Editor,

Active genes exist in the euchromatic regions of centromeres across several species (Nagaki et al. 2004; Wu et al. 2011; Lian et al. 2024). Until now, there have been limited studies addressing the functions of the active genes located in the centromeric region of plants. Previously, we identified several putative active genes within the centromeric region of rice (*Oryza sativa* L.) chromosome 8 (Xue et al. 2022). Among them, LOC_Os08g21700 encodes a chloroplast precursor, homologous to the *chloroplast and leaf defective* (*DCL*) gene of tomato and the *AtDCL* gene of Arabidopsis, and is named *OsDCL* (Fig. 1A). In tomato, *DCL* is essential for plastid ribosomal RNA and functions at an early stage of chloroplast development (Keddie et al. 1996; Bellaoui et al. 2003). In Arabidopsis, aberrant expression of the *AtDCL* results in yellowing leaves, sterile ﬂowers, anomalous cotyledons, and even death (Bellaoui and Gruissem 2004). Nevertheless, the functional roles of *OsDCL* in rice remain unelucidated.

**Figure 1.**
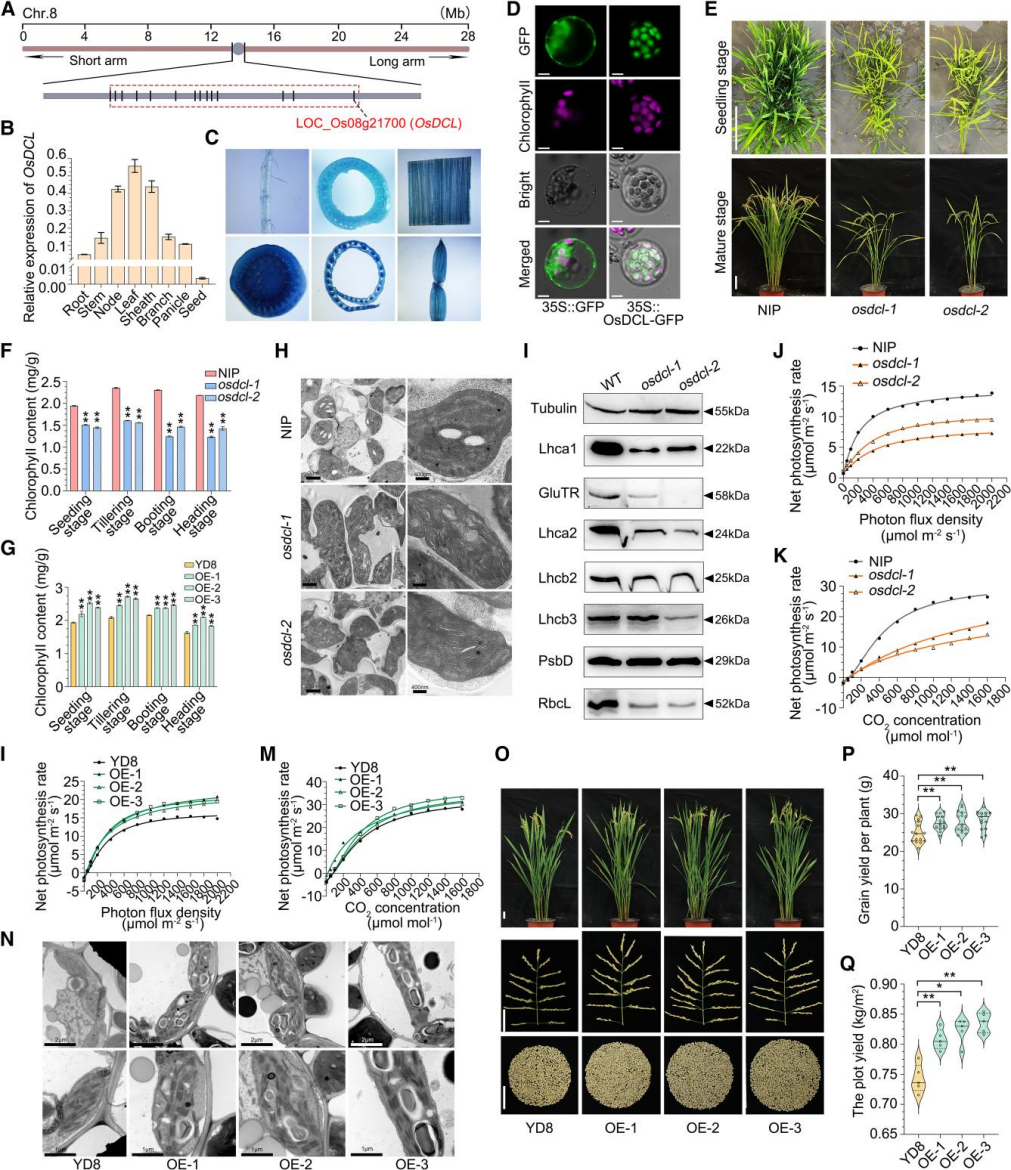
*OsDCL*, a gene located in centromeric region, regulates plant development and grain yield in rice. **A)** The location of *OsDCL* in the centromeric region in rice chromosome 8. Adapted from our previous study (Xue et al. 2022). **B)** Relative expression levels of *OsDCL* in different tissues detected by RT-qPCR (*n* = 3). **(C)** GUS activities in root, stem, leaf, node, sheath, and spikelet hulls. **D)** Subcellular localization analysis of OsDCL protein. 35S::GFP was used as the control. Scale bars, 5 μm. **E)** Plant morphology of NIP and *osdcl* mutants at seedling and mature stages. Scale bars, 10 cm. **F)** Total chlorophyll content in the leaves of NIP and *osdcl* mutants at different stages (*n* = 3). **G)** Total chlorophyll content in the leaves of YD8 and overexpression lines of *OsDCL* at different stages (*n* = 3). **H)** TEM images of leaf chloroplasts in NIP and *osdcl* mutants. Scale bars, 2 μm (left); Scale bars, 400 nm (right). **I)** Western-blot analysis of photosynthesis-related proteins in NIP and *osdcl* mutants. **J, K)** Light and CO_2_ response curves in the flag leaves of NIP and *osdcl* mutants. **L, M)** Light and CO_2_ response curves in the flag leaves of YD8 and overexpression lines of *OsDCL*. **N)** TEM images of leaf chloroplasts in YD8 and OE lines. Scale bars, 2 μm (top row); Scale bars, 1 μm (bottom row). **O)** Morphology of plants, panicles, and entire grains harvested from single plants of YD8 and OE lines. Scale bars, 5 cm. **P)** Comparisons of grain yield per plant between YD8 and OE lines (*n* ≥ 15). **Q)** Comparisons of plot yield between YD8 and OE lines (*n* = 5). The *osdcl-1* and *osdcl-2* lines are two independent knockout mutants of *OsDCL* generated in the NIP background, while OE-1, OE-2, and OE-3 are the three distinct overexpression lines of *OsDCL* developed in the YD8 background. Data are shown as Means ± SDs. Statistical differences were determined using Student's *t*-test: *, *P* < 0.05; **, *P* < 0.01. All primers used are summarized in Supplementary Table S1.

We initially investigated the expression pattern of *OsDCL*. RT-qPCR analysis showed that *OsDCL* was constitutively expressed in all tested tissues, with the highest expression in leaf, sheath, and node, and the lowest expression in seed (Fig. 1B). Promoter-driven GUS reporter analysis showed a largely similar expression pattern (Fig. 1C). Transient expression of the 35S::OsDCL-GFP construct in rice protoplasts demonstrated that OsDCL was localized in the chloroplasts (Fig. 1D). To further uncover the biological function of *OsDCL* in rice, we utilized CRISPR/Cas9 technology to generate knockout mutants of *OsDCL* in the Nipponbare (NIP) background (see Supplementary Materials and Methods). Two homozygous mutants (named *osdcl-1* and *osdcl-2*), carrying a single base pair deletion and a single base pair insertion in the target sequence (Supplementary Fig. S1), respectively, were subjected to detailed analysis. Throughout the entire growth period, the *osdcl-1* and *osdcl-2* mutants exhibited a yellow–green leaf phenotype (Fig. 1E). The mutants showed significantly reduced chlorophyll contents at seedling, tillering, booting, and heading stages (Fig. 1F). We conducted RNA-seq analysis using 2-week-old seedlings of *osdcl-1* and NIP. Compared with NIP, the *osdcl-1* mutant displayed significant alterations in gene expression profile, comprising 1,577 upregulated and 1,506 downregulated genes (Supplementary Fig. S2A). Gene ontology enrichment analysis showed that the 1,506 downregulated genes were significantly enriched in cellular component classes associated with chloroplast, plastid and thylakoid parts (Supplementary Fig. S2B). Furthermore, the expression of several key genes in chlorophyll synthesis, photosynthesis, and chloroplast development pathway were decreased in the *osdcl* mutants (Supplementary Fig. S3A). Transmission electron microscopy (TEM) showed that knockout of *OsDCL* led to abnormally developed chloroplasts, characterized by disorganized thylakoid membrane structure and abnormal grana configurations (Fig. 1H). Western-blot analysis indicated a substantial reduction in the abundance of several critical photosynthetic proteins, specifically Lhca1, GluTR, Lhca2, Lhcb3, and RbcL, in the *osdcl* mutants (Fig. 1I). Because chlorophyll content and chloroplast development are closely related to rice photosynthesis (Wang et al. 2015; Zhang et al. 2023), we compared the photosynthetic rates between NIP and the *osdcl* mutants. Analysis of the photosynthetic light response and CO_2_ response curves demonstrated that the net photosynthetic rates of *osdcl-1* and *osdcl-2* were substantially lower than that of NIP (Fig. 1J, K). From the seedling stage onward, the *osdcl* mutants displayed pronounced growth inhibition, manifested primarily by reduced tiller formation (Supplementary Fig. S4A, B). At the mature stage, the *osdcl* mutants exhibited significantly reduced grain size, plant height, panicle number, grain number per panicle, and grain yield per plant compared to NIP (Supplementary Fig. S4C-L). These results indicate that the loss-of-function of *OsDCL* seriously impacts chloroplast development and photosynthetic process, subsequently resulting in a substantial hindrance in plant growth and a consequential decrease in yield.

To further evaluate the genetic effects of *OsDCL* in rice, we constructed transgenic lines overexpressing *OsDCL* in the *japonica* variety Yandao8 (YD8) background, which is a high-yield variety that was once widely cultivated. Three independent overexpression lines (OE-1, OE-2, and OE-3) with significantly increased *OsDCL* expression levels were used for further analysis (Supplementary Fig. S5). Compared to YD8, chlorophyll content in the leaves of the three OE lines increased by 10.0% to 13.4% at seedling, tillering, booting and heading stages (Fig. 1G). The expression of several key genes in chlorophyll synthesis, photosynthesis, and chloroplast development pathway were increased in the OE lines (Supplementary Fig. S3B). Additionally, the net photosynthetic rates were obviously increased in the three OE lines (Fig. 1L, M). TEM analysis showed that the chloroplasts of the OE lines contained regularly distributed grana (Fig. 1N).

In field conditions, the three OE lines demonstrated greater growth potential. Compared to YD8, the OE lines had more tillers (Supplementary Fig. S6A, B). While the OE lines showed significantly increased grain length and width compared to the wild-type, their grain thickness was reduced, accounting for the observed decrease in grain weight (Supplementary Fig. S6C-H). Compared to YD8, the plant height, panicles number per plant and grain number per panicle of the OE lines were also significantly increased (Fig. 1O; Supplementary Fig. S6I-K). At maturity, compared to YD8, the *OsDCL* overexpression lines showed increased biomass yield per plant (Supplementary Fig. S6L). Moreover, grain yield per plant of the three OE lines increased by 10.6% to 13.5% (Fig. 1O, P). These findings were corroborated by field plot trials, where the three OE lines demonstrated yield increases of 8.8%, 10.9%, and 12.6% per plot, respectively (Fig. 1Q). However, three OE lines exhibited reduced appearance quality (Supplementary Fig. S7). These data indicate that overexpression of *OsDCL* promotes plant growth and significantly increases grain yield production in rice.

To date, functional studies on actively transcribed genes within rice centromeric regions have made limited progress (Xie et al. 2023). In this study, we characterized *OsDCL* as a functionally essential gene residing within the centromeric region of chromosome 8 in rice. Our study demonstrates that *OsDCL* mutations induce severe chloroplast ultrastructural defects, leading to reduced chlorophyll content and impaired photosynthetic capability, ultimately stunting plant growth. Conversely, overexpression of *OsDCL* elevates chlorophyll accumulation, enhances photosynthetic performance, and boosts grain yield. These findings provide evidence that transcriptionally active genes in centromeric regions can play functional roles, suggesting that centromeres may not be entirely genetically silent. Given the centromeric localization of *OsDCL*, future investigations should systematically examine whether its regulatory mechanisms exhibit fundamental divergence from those governing euchromatic gene expression.

## Supplementary Material

kiaf500_Supplementary_Data

## Data Availability

The data that support the ﬁndings of this study are available on request from the corresponding author. The data are not publicly available due to privacy or ethical restrictions.

## Supplementary data

The following materials are available in the online version of this article.

Additional **Supplementary information** may be found online in the **Supplementary Information** section at the end of the article.

**Supplementary Materials** and **methods**.

**Supplementary Figure S1.** Identification of the *osdcl* mutants.

**Supplementary Figure S2.** RNA-seq analysis of 2-week-old *osdcl-1* and NIP rice seedlings.

**Supplementary Figure S3.** Heatmap of expression levels of several essential genes for chlorophyll synthesis, photosynthesis and chloroplast development in different transgenic lines.

**Supplementary Figure S4.** Agronomic trait analysis of the *osdcl* mutants.

**Supplementary Figure S5.** Relative expression levels of *OsDCL* in the overexpression lines.

**Supplementary Figure S6.** Agronomic trait analysis of overexpression lines of *OsDCL*.

**Supplementary Figure S7.** The milling quality and appearance quality analysis of overexpression lines of *OsDCL*.

**Supplementary Figure S8.** Structural diagrams of all the constructs used in this study.

**Supplementary Table S1** Primers used in this paper.
