# Supplementary material for: The centromeric gene OsDCL plays essential roles in plant development and yield production in rice
Source: Plant Physiol. 2025 Oct 29;199(2):kiaf500. doi: 10.1093/plphys/kiaf500 (PMC12569755; doi:10.1093/plphys/kiaf500)
Supplement: kiaf500_Supplementary_Data [file kiaf500_supplementary_data.zip › Supplementary Data.pdf]

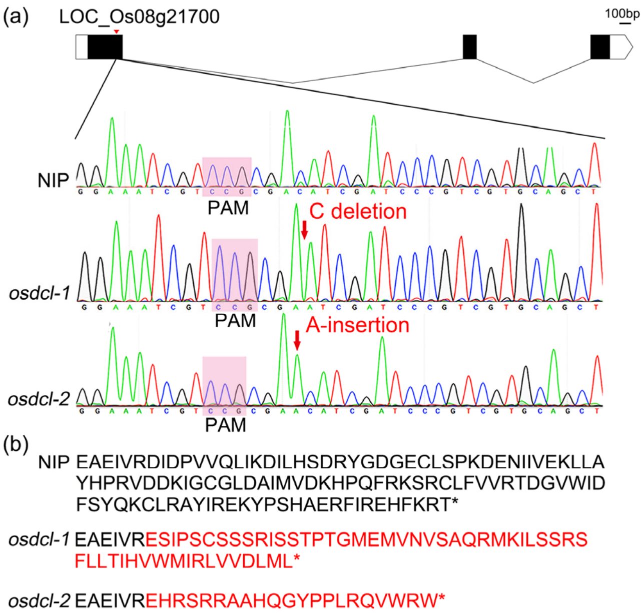

(b)

NIP EAEIVRDIDPVVQLIKDILHSDRYGDGECLSPKDENIIVEKLLA
YHPRVDDKIGCGLDAIMVDKHPQFRKSRCLFVVRTDGVWID
FSYQKCLRAYIREKYPSHAERFIREHFKRT*

osdcl-1 EAEIVRESIPSCSSSRISSTPTGMEMVNVSAQRMKILSSRS
FLLTIHVWMIRLVVDLML*

osdcl-2 EAEIVREHRSRRAAHQGYPPLRQVWRW*

Supplementary Figure S1. Identification of the *osdcl* mutants. (a) Schematic diagram showing the targets and mutated sites of *OsDCL* by CRISPR/Cas9-based genome editing. The sequence of the first exon of the LOC_Os08g21700 (*OsDCL*) gene was selected as the target site of sgRNA. The *osdcl-1* mutant exhibited a single-nucleotide deletion within the target region, whereas a nucleotide insertion was identified at the corresponding locus in *osdcl-2*. Red arrows indicate mutated sites and pink shading indicates PAM sequences. (b) The amino acid differences of OsDCL protein between NIP and *osdcl* mutants. Both mutants generated premature termination codons, resulting in truncated polypeptide products.

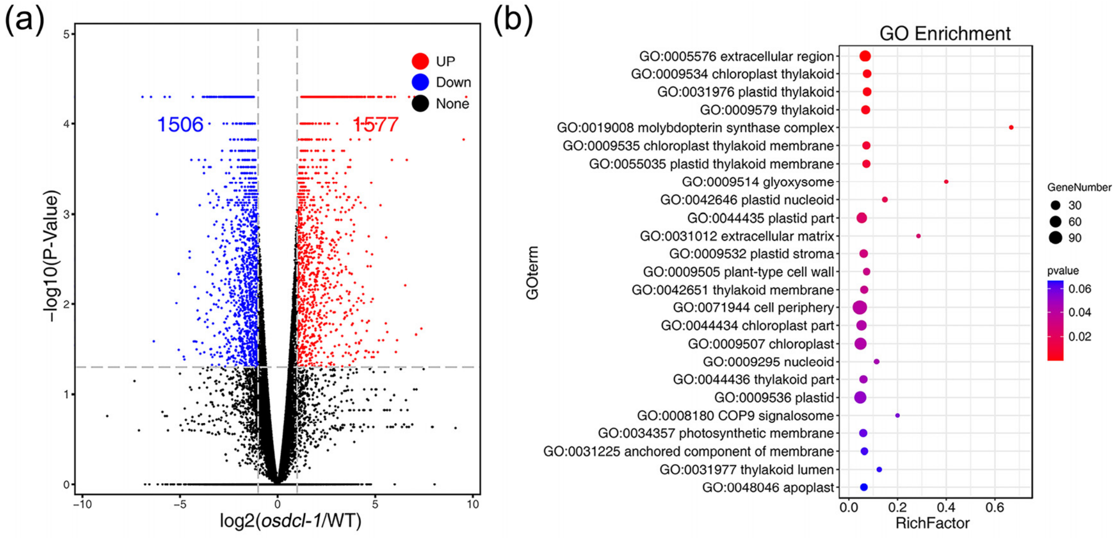

Supplementary Figure S2. RNA-seq analysis of 2-week-old *osdcl-1* and NIP rice seedlings. (a) Volcano plot depicting differentially expressed genes (DEGs) in NIP and *osdcl-1* mutant. Red dots represent genes expressed at higher levels in *osdcl-1* mutant while blue dots represent genes with lower expression levels in *osdcl-1* mutant. Y-axis denotes −log10 P values while X-axis shows log2 fold change values. (b) Gene ontology (GO) enrichment analysis of 1,506 down-regulated genes in the biological process.

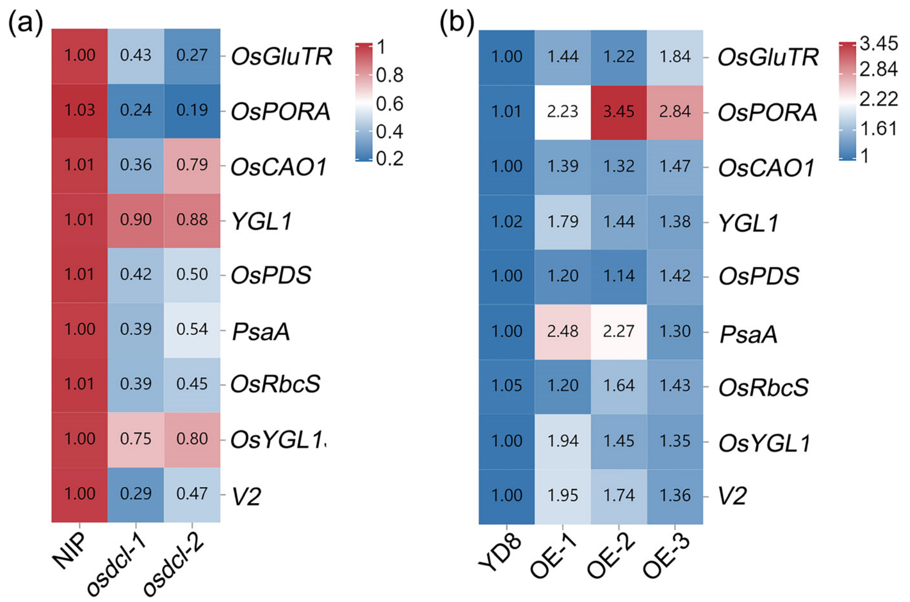

Supplementary Figure S3. Heatmap of expression levels of several essential genes for chlorophyll synthesis, photosynthesis and chloroplast development in different transgenic lines. (a) Relative expression levels of chlorophyll synthesis, photosynthesis and chloroplast development related genes in NIP and *osdcl* mutants. (b) Relative expression levels of chlorophyll synthesis, photosynthesis and chloroplast development related genes in in YD8 and OE lines. The *osdcl-1* and *osdcl-2* lines are two independent knockout mutants of *OsDCL* generated in the NIP background, while OE-1, OE-2 and OE-3 are the three distinct overexpression lines of *OsDCL* developed in the YD8 background. RT-qPCR was used for expression analysis. Each row represents a gene, and each column represents a sample. The expression level of the rice ubiquitin gene (*UBQ*) was determined as a control. Values are means ± SDs of three independent experiments.

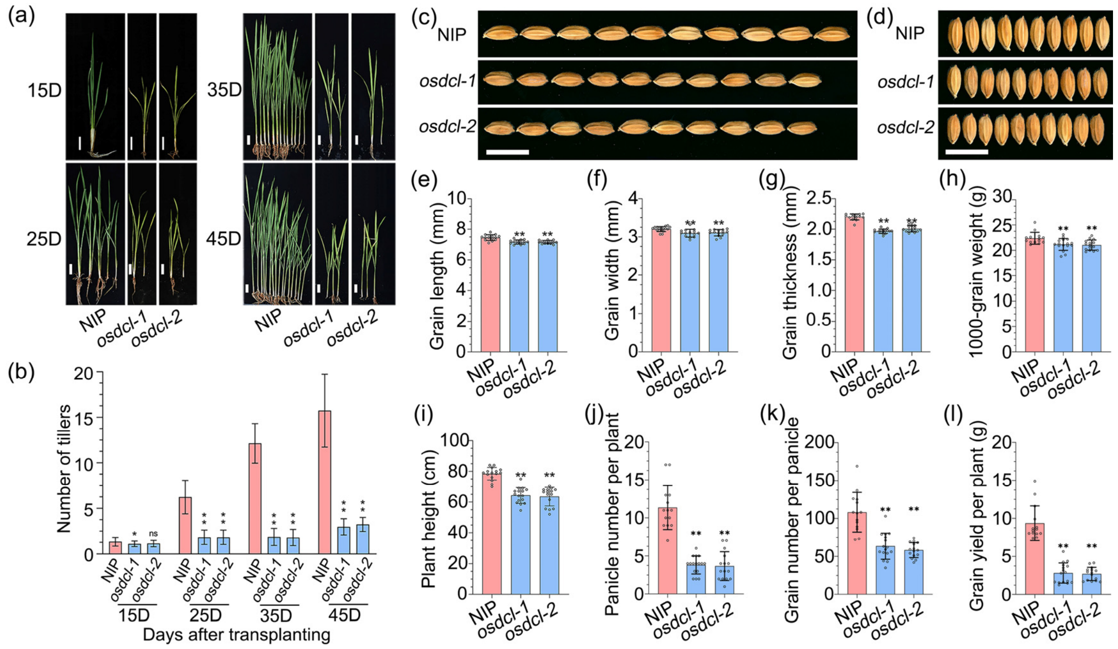

Supplementary Figure S4. Agronomic trait analysis of the *osdcl* mutants. (a) Morphology of tillers of NIP and *osdcl* mutants at 15, 25, 35, 45 days after transplanting. All the scale bars, 5 cm. (b) Comparisons of tiller numbers of NIP and *osdcl* mutants at 15, 25, 35, 45 days after transplanting. The data are given as the means ± SDs (n = 30). (c-d) Grain morphology of NIP and *osdcl* mutants. All the scale bars, 1 cm. (e-l) Comparisons of grain length (e), grain width (f), grain thickness (g), 1000-grain weight (h), plant height (i), panicle number per plant (j), grain number per panicle (k), and grain yield per plant (l) between NIP and *osdcl* mutants. The data are given as the means ± SDs (n ≥ 15). Student's t-test: *, $P < 0.05$; **, $P < 0.01$; ns, not significant.

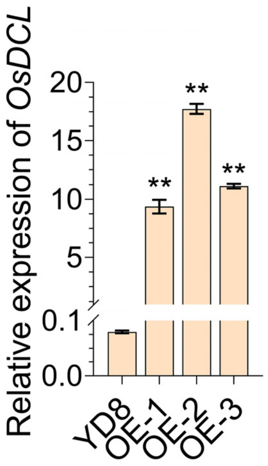

Supplementary Figure S5. Relative expression levels of *OsDCL* in the overexpression lines. OE-1, OE-2 and OE-3 are the three distinct overexpression lines of *OsDCL* developed in the YD8 background. RT-qPCR was used for expression analysis. The expression level of the rice ubiquitin gene (*UBQ*) was determined as a control. Values are means ± SDs of at least three independent experiments. Student's t-test: *, $P < 0.05$; **, $P < 0.01$.

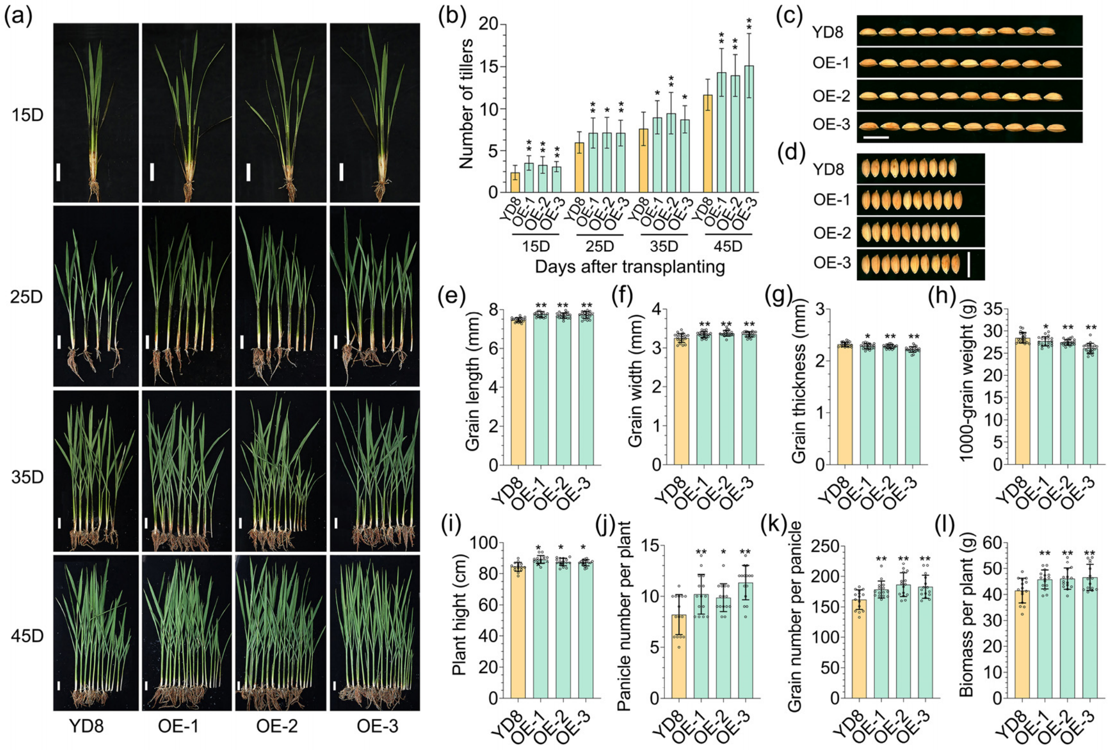

Supplementary Figure S6. Agronomic trait analysis of overexpression lines of *OsDCL*. (a) Tillers of YD8 and *OsDCL*-overexpressing lines at 15, 25, 35 and 45 days after transplanting. All the scale bars, 5 cm. (b) Tiller number of YD8 and *OsDCL*-overexpressing lines at 15, 25, 35 and 45 days after transplanting (n = 30). (c-d) Grain morphology of YD8 and *OsDCL*-overexpressing lines. All the scale bars, 1 cm. (e-l) Comparisons of grain length (e), grain width (f), grain thickness (g),1000-grain weight (h), plant height (i), panicle number per plant (j), grain number per panicle (k) and biomass per plant (l) between YD8 and *OsDCL*-overexpressing lines. OE-1, OE-2, and OE-3 are the three overexpression lines of *OsDCL*. The data are given as the means ± SDs (n ≥ 15). Student's t-test: *, $P < 0.05$; **, $P < 0.01$.

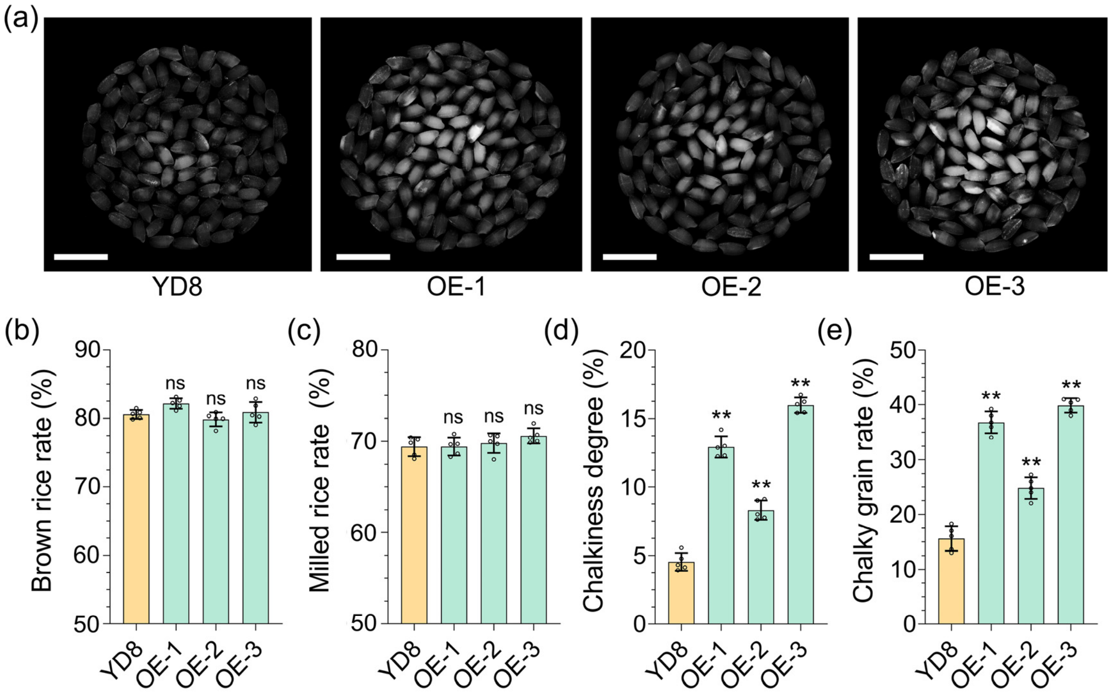

Supplementary Figure S7. The milling quality and appearance quality analysis of overexpression lines of *OsDCL*. (a) The appearance of milled rice of YD8 and *OsDCL*-overexpressing lines. Scale bars, 1 cm. (b-e) Comparison of brown rice rate (b), milled rice rate (c), chalkiness degree (d) and the chalky grain rate (e) between YD8 and *OsDCL*-overexpressing lines. OE-1, OE-2, and OE-3 are three overexpression lines of *OsDCL*. The data are given as the means ± SDs (n = 5). Student's t-test: *, $P < 0.05$; **, $P < 0.01$.

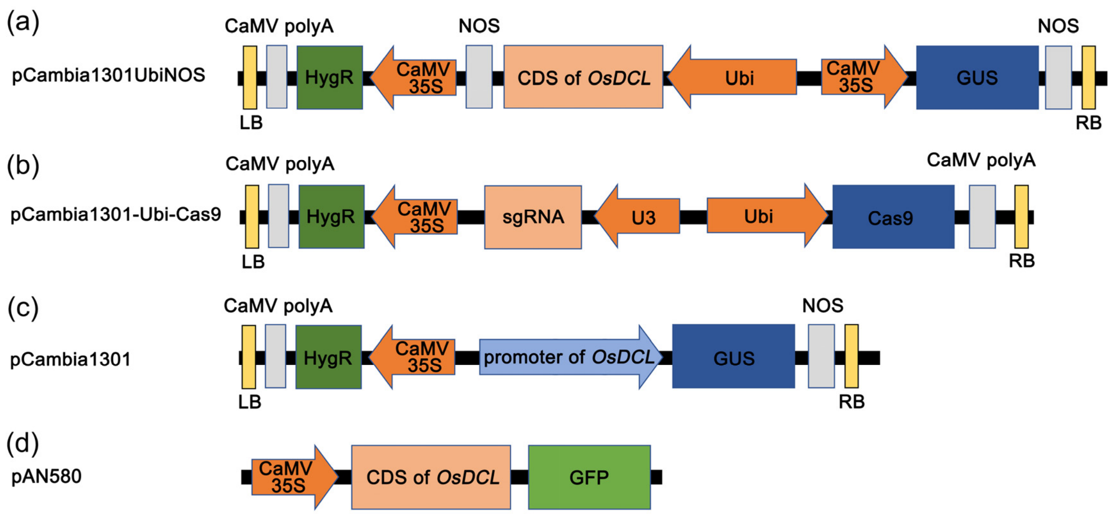

Supplementary Figure S8. Structural diagrams of all the contructs used in this study. (a) pCambia1301UbiNOS was used for the creation of overexpression contruct, in which gene expression was driven by the Ubiquitin promoter from maize. (b) pCambia1301Ubi-Cas9 was employed for generation of CRISPR/Cas9 mutants, in which the Cas9 destination vector was driven by the maize Ubiquitin promoter, and sgRNA expression was driven by the U3 promoter. (c) pCambia1301 was used for the construction of GUS fusion vector, in which GUS gene expression was driven by the *OsDCL* promoter. (d) pAN580 was used for the subcellular localisation, in which the expression of OsDCL protein was driven by the CaMV35S promoter.

Supplementary Table S1 Primers used in this paper.

Primer name	Forward primer (5'-3')	Reverse primer (5'-3')	Purpose
DCL-sg	GGCAGCACGACGGGATCGATGTCG	AAACCGACATCGATCCCGTCGTGC	CRISPR construct
DCL-pCambia1301UbiNOS	AAAACTAGTATGGCCCTGGCAGCGGCACTCG	AAAGGTACCTCAGGTTCGCTTGAAATGTTCTCGTA	Overexpressing construct
DCL-pCambia1301	AAAGGTACCTGGTGTAGCGTTGATCAGTGGAGA	AAACCATGGGCGGCGGGAGGGTGACTTGCT	GUS construct
dcl-detection	GGCAGAGAAATGGCCAAATGC	GTCACTCGTCACTTCGTCAGT	Mutant detection
DCL-pAN580	AGGACCGGTCCCGGGGGATCCATGGCCCTGGCAGCGGCA	GCCCTTGCTCACCATGGATCCTCAGGTTCGCTTGAAATGTTCTC	Subcellular localization construct
OsDCL- qPCR	GGTGAATGTCTCAGCCCAAAG	CCATCCGTGCGAACAACA	RT-qPCR
OsGluTR- qPCR	CGCTATTTCTGATGCTATGGGT	TCTTGGGTGATGATTGTTTGG	RT-qPCR
OsPORA- qPCR	ATCATCCTCGGCTCCATCAC	GCATCGTCAGCATGTTACAGA	RT-qPCR
OsCAO1- qPCR	GATCCATACCCGATCGACAT	CGAGAGACATCCGGTAGAGC	RT-qPCR
YGL1- qPCR	TGGATATGTGTAGGAGCGATTG	CCACCTGAGGAATTGTGAGTC	RT-qPCR
OsPDS- qPCR	TGGATACTGGCTGCCTGTCA	GTGCTTGGATGCTGCTACTTG	RT-qPCR
PsaA- qPCR	GCGAGCAAATAAAACACCTTTC	GTACCAGCTTAACGTGGGGAG	RT-qPCR
OsRbcS- qPCR	TCCGCTGAGTTTTGGCTATTT	GGACTTGAGCCCTGGAAGG	RT-qPCR
OsYGL138- qPCR	AAGAGTTGTATGCTGGTCGCT	TTGGCTTCTGTTCCTTCTGAGT	RT-qPCR
V2- qPCR	GGAGTTCCTCACGATGATTGAA	GGTTGCTGCTCCTTGAATGT	RT-qPCR
OsUBQ- qPCR	AACCAGGCTGAGGCCCAAGA	ACGATTGATTTAACCAGTCCATGA	RT-qPCR
