# Supplementary material for: The centromeric gene OsDCL plays essential roles in plant development and yield production in rice
Source: Plant Physiol. 2025 Oct 29;199(2):kiaf500. doi: 10.1093/plphys/kiaf500 (PMC12569755; doi:10.1093/plphys/kiaf500)
Supplement: kiaf500_Supplementary_Data [file kiaf500_supplementary_data.zip › Supplementary Materials and methods.pdf]

Materials and methods

Plant materials and growth conditions

Two *japonica* cultivars of rice, Nipponbare (NIP) and Yandao 8 (YD8), were used in this study. Two homozygous knock-out mutants (*osdcl-1* and *osdcl-2*) in the NIP background were generated using the CRISPR/ Cas9 system. We selected three homozygous OE lines (OE-1, OE-2 and OE-3, T_4 generation) in the YD8 background for the phenotypic analysis. The wild-type and transgenic lines were grown in experimental fields (E 119°25′/N 32°23′) of Yangzhou University during the normal growing seasons. Each line was grown in a plot containing 40 individuals. The distance between the individuals within a row was 13.3 cm, and the distance between the rows was 23.3 cm. The experimental site for plot yield assessment comprised over 500 individual plants.

Yield and yield component determinations

Agricultural traits of wild-type and transgenic lines were investigated at maturity stage in 2024. Plant height was assessed from the top of the tallest panicle to the ground level. The panicles from the main stems were chosen for counting the number of grains per panicle. The number of effective panicles with 10 or more grains represented the panicle number per plant. 1000-grain weight were measured after the grains had been harvested and stored at 37°C for at least one week. The grain yield per plant and per plot was assessed by harvesting seeds from all panicles. The grains of the plants grown in 1 m^2 were harvested for plot yield measurement. Seeds were dried at 37°C for one week before weighing. The border plants in each plot were removed to mitigate margin effects during sampling, measurements, and final yield analysis.

Vector construction and transformation

To generate the OE construct, the full-coding region of *OsDCL* was amplified from NIP complementary DNA (cDNA) and inserted into pCambia1301UbiNOS, which was driven by a maize ubiquitin promoter. In each generation, the hygromycin resistance of leaves was used as the selection criterion to determine positive transgenic plants and homozygous lines.

To generate the CRISPR/Cas9 mutants, a single-guide RNA (sgRNA) targeting the 1nd exon of the *OsDCL* gene was selected. The final fragment was inserted into a CRISPR/Cas9 system, in which the Cas9 destination vector was driven by the maize ubiquitin promoter for expression

in rice and sgRNA expression was driven by the U3 promoter. Cetyltrimethylammonium bromide method was used to extract genomic DNA of transgenic lines. The genomic region surrounding the CRISPR target site for *OsDCL* was amplified by PCR, and the segment was sequenced to screen for mutants.

The 1,940-bp promoter region of *OsDCL* was amplified for expression pattern analysis. The amplification product was subcloned into pCambia1301 to generate the *proOsDCL*::GUS fusion construct.

Structural diagrams of all the contructs had been shown in Figure S8. All of the constructs were independently transferred into receptors using *Agrobacterium tumefaciens*-mediated transformations. The primer sequences used for vector construction are listed in Supplementary Table S1.

Quantification of chlorophyll content

In a low-light or light-avoidance setting, 0.1 g of rice leaf tissue was combined with 1 ml of distilled water and thoroughly ground with quartz sand. The resultant grinding products were completely extracted using 10 ml of 80% acetone until the tissue residue appeared entirely white. The absorbance values of the extracts at 663 and 645 nm were recorded as A_{663} and A_{645}, respectively, and the concentrations of chlorophyll a, chlorophyll b, and total chlorophyll were calculated using the specified equations. Total chlorophyll content (mg/g fresh weight) = $(20.21 \times A_{645} + 8.02 \times A_{663}) \times V \times D \div m \div 1000$; V = 10 mL; D = dilution factor; m = sample mass in grams.

Transmission electron microscopy (TEM)

Cotyledons from 14-day-old seedlings were subjected to TEM analysis by being fixed in a solution containing 2.5% (v/v) glutaraldehyde and 2% (v/v) paraformaldehyde, diluted in 0.1 M PBS buffer (pH 7.4), at 4 °C overnight. Following three washes with 0.1 M PBS buffer, the samples were stained with 1% osmium tetroxide (w/v) in the same buffer for 3 hours at 4 °C, and subsequently underwent three more washes with 0.1 M PBS buffer. Dehydration samples were conducted using a series of graded ethanol at 4 °C, followed by ultimate dehydration with 100% acetone augmented with anhydrous sodium sulfate, and fresh resin was employed for embedding. Following polymerization, ultrathin sections (70 nm) were produced utilizing a

Leica EM ultramicrotome. Post-staining was conducted utilizing uranyl acetate and lead citrate. Samples were examined using a TEM (Tecnai G2 F30 S-TWIN, FEI, USA).

Leaf photosynthesis measurement

Leaf photosynthesis was measured in flag leaves at the full heading stage with a portable photosynthesis system (LI-6800, Li-Cor, USA). Measurement was conducted on clear days between 0900 and 1200 h. The air temperature in the sample chamber was controlled according to the ambient temperature. For the light response curve, the reference CO_2 concentration was fixed at 395 μmol mol-1 and the light intensity gradient was set at 2200, 2000, 1800, 1600, 1400, 1200, 1000, 800, 600, 400, 200, and 0 μmol m^{-2} s^{-1}. For the CO_2 response curve, the light intensity was fixed at 2000 μmol m^{-2} s^{-1} and the reference CO_2 concentration gradients were set at 200, 400, 600, 800, 1000, 1200, 1400, 1600, and 1800 μmol mol^{-1} s^{-1}.

RNA extraction and quantitative reverse transcription PCR (qPCR)

Total RNA was extracted from each tissue using a Simple Total RNA kit (RC411-01, Vazyme, China) according to the manufacturer's instructions and treated with DNase to remove any genomic DNA. Approximately 1 μg of total RNA was used for cDNA synthesis with a FastQuant RT kit (R312-02, Vazyme, China). Expression patterns were analyzed using RT-qPCR. The RT-qPCR assay was performed on an ABI real-time PCR system (ViiA7, Applied Biosystems, USA) following the recommended protocol of the manufacturer with the ChamQ SYBR Color qPCR Master Mix (Q511-02, Vazyme, China). The rice ubiquitin gene (LOC_Os03g13170) was used as an internal control. The primer sequences used for the RT-qPCR are listed in Supplementary Table S1.

Histochemical GUS analysis

Fresh samples from *proOsDCL*::GUS transgenic plants at the heading stage were incubated in GUS solution containing 50 mM phosphate buffer (pH 7.2), 0.5% (v/v) Triton-X100, 5 mM potassium ferricyanide, 5 mM potassium ferrocyanide and 2 mM 5-bromo-4-chloro-3-indolyl-β-D glucuronide at 37°C for 24 h. The reaction was stopped by adding ethanol, and then, the samples were treated with fresh 70% ethanol several times until the plant tissues were mostly discolored. Subsequently, the samples were examined using a microscope (DM1000, Leica,

Germany) and photographed with a digital camera.

RNA-seq analysis

RNA samples were obtained from 14-day-old seedling leaves. RNA sequencing was performed by Biomarker Technologies Corporation (Beijing, China). Trimmomatic (v0.38) was used to remove splicing artifacts and low-quality reads from the original sequencing data. The filtered clean reads were aligned to the rice genome [Nionaru, MSU v7.0 using Hisat2 (v2.1.0)]. Only uniquely mapped reads (Phred Quality Score > 20) were used for differential expression analysis, and Cufflinks (v2.2.1) was used to calculate gene expression levels (FPKM). Genes with a P-value < 0.05 and log2(Fold change) = 1 were defined as differentially expressed genes. All statistical analyses were performed using R (v4.0.2). RNA-seq data supporting the results of the study are available in the NCBI Sequence Read Archive (SRA) repository, under NCBI SRA (Accession No. PRJNA1213707). The functional category analysis of the DEGs was performed using g:Profiler with a false discovery rate < 0.05 (https://biit.cs.ut.ee/gprofiler/gost). Enrichment results were clustered and visualized using REVIGO (http://revigo.irb.hr/).

Isolation of rice protoplasts and plasmid transformation

Plasmid transformation of rice protoplasts was performed using polyethylene glycol (PEG 4000) mediation. Stem tissues were harvested from 15-day-old rice plants and used as the source material for protoplast isolation. The tissues were digested in an enzyme solution containing 0.6 M mannitol, 1.6% cellulose R-10, 0.75% Macerozyme R-10, 0.1% BSA, 10 mM 2-(N-morpholino)ethanesulfonic acid (MES), 1 mM $CaCl_2$, and 0.4% β-mercaptoethanol. The isolated protoplasts were then washed with W5 solution (154 mM NaCl, 125 mM $CaCl_2 \cdot 2H_2O$, 5 mM KCl, 5 mM glucose, 2 mM MES). Plasmid DNA was mixed with the prepared rice protoplasts, followed by gentle addition and mixing of an equal volume of 40% (w/v) PEG solution (PEG 4000, 0.1 M $CaCl_2 \cdot 2H_2O$, 0.2 M mannitol). After incubating the mixture under dark conditions at 28 °C for 12 hours, fluorescence was detected using confocal microscope (LSM 710, Zeiss, Germany)

Subcellular localization

To determine the subcellular localization of the OsDCL protein, the full-length coding region

of *OsDCL* was amplified and linked into the pAN580 vector, in which is driven by a constitutively expressed CaMV35S promoter. The fusion construct 35S::OsDCL-GFP was transferred into rice NIP protoplasts by polyethylene glycol-mediated transformation, and the empty pAN580 (35S::GFP) was used as a control. After 12h in the dark, the subcellular localizations of the recombinant proteins were observed using the above-mentioned confocal microscope.

Western blot analysis.

Total protein was extracted from leaf of 2-week-old rice seedings with 200 μL extraction buffer containing 20 mM Tris (pH 7.5), 100 mM NaCl, 2.5 mM $MgCl_2$, 1 mM EGTA, 1 mM DTT and 1mM PMSF. After denaturation, protein were separated by SDS-PAGE and transferred to PVDF membranes (IPVH00010, Millipore, USA). After blocking with 5% skim milk in TBST buffer (20 mM Tris/HCl, pH 7.6, 137 mM NaCl, 0.1% Tween), membranes were incubated with primary antibodies raised against Lhca1 (AS01-005), GluTR (AS10-689), Lhca2 (AS01-006), Lhcb2 (AS01-003), Lhcb3 (AS01-002), PsbD (AS06-146) and RbcL (AS03-037) (Agrisera, Sweden) at the dilutions recommended by the supplier, followed by incubation with secondary antibody (goat anti-rabbit, CW0103S, China) at a dilution of 1:10,000. An antibody against Tubulin (MA1-80017, ThermoFisher, USA) was used as a loading control. Membranes were imaged using a chemiluminescence imager device (5200Multi, Tanon, China) after incubation in ECL solution (A38556, ThermoFisher, USA) for 2 min at room temperature.

Statistical analyses

Data are presented as the means ± SDs (error bars indicate the standard deviations of the means). Statistical analyses were carried out using Excel (Microsoft, USA) and GraphPad Prism software (GraphPad, USA). The differences were determined using Student's *t*-test (*, $P < 0.05$; **, $P < 0.01$; and ns, not significant).
